# Pneumonia Miasmatica

**Published:** 1850-03

**Authors:** J. J. Lescher

**Affiliations:** Mount Carmel, Illinois


### ARTICLE VIII.

*Pneumonia Miasmatica.*—By J. J. Lescher, M.D., of Mount Carmel, Illinois.

In using the term, *Pneumonia Miasmatica*, I do not wish to lay myself obnoxious to the charge of neologism, for, on the contrary, I design it only to answer my present purpose, that of offering a few hasty and crude suggestions relative to Pneumonitis, as it usually prevails in malarious localities.

The reflections which, in the course of this communication, I may offer, have been suggested by occasional notices found in medical periodicals, particularly those of Western growth; by frequent conversation had with practitioners, and latterly, by having read in your Journal, two communications on the subject, by Drs. Howland and Banks.

In arriving at a correct knowledge of treating diseases skilfully, and in accordance with written experience, the best means through which such a knowledge is to be acquired, is a definite, and if I may be allowed the expression, a uniform and unambiguous medical nomenclature. Words or terms are mere expressions of ideas; and unless the term implies a certain and definite idea, its use had better be discarded, as tending only to bewilder and confuse the mind of the reader.

We hear of the prevalence of "winter fever"—of "lung fever;" of pneumonia. Physicians tell us of the happy effects of certain remedies in the disease known by the above appellations. Discrepancies of opinions and practice are wonderfully frequent.

Such a state of facts is greatly to be regretted, inasmuch as it tends rather to obscure, than to enlighten the understanding of the anxious inquirer. This perplexity can be obviated by the use of terms expressive of the pathological conditions we design to convey.

In speaking of 'winter fever,' we should expect to be understood as meaning pneumonia—purely an inflammation of the lung, such as is understood by all authors, and as requiring the use of antiphlogistic means towards its removal.

In our efforts to subdue *inflammation* of the lungs, I cannot, for the life of me, comprehend the possibility of destroying the disease in the short space of "two or three days," unless it be by the opportune interposition of a copious depletion, and by that *alone*, if the general notion of inflammation is correct. Dr. John Mason Good gives his opinion that, in the most favorable cases, "the violence of the disease diminishes on or before the seventh day." He seems to know of nothing possessed of such astonishing virtues in cutting short the malady, unless the loss of blood—concerning which, he says: "it has occasionally proved critical, and carried off the disease in a few days." In continuation, he says: "The best, the easiest, and even the natural cure of peripneumony, (pneumonia, or 'winter fever,') is expectoration, which, hence, ought to be excited, and encouraged by all means in our power. It forms the *optima crisis* of Stoll."

A late writer, and one whose opinion might be regarded as more to be relied on, because forsooth he *is* a late author, says: "When it (winter fever) goes by resolution, some very evident evacuation almost always attends it; such as a great flow of urine, &c., but the evacuation which most frequently terminates the complaint, and which does it with the *greatest effect*, is a free and copious expectoration of thick white, or yellow matter, slightly streaked with blood; and by this the disease is carried off gradually in the course of ten or twelve days."

The writers of the above named articles,--unintentionally, I think—seem to carry out the idea, that "the medicine"---the *optimum remedium* in pneumonitis, is, par excellence, *Sulphas Quiniæ*. Now for my part, and I believe the experience of the mass of Western, or if you please, malarious practice, will bear me out in my opinion, I cannot subscribe to the correctness of any such conclusion, nor even an application of it.---Seldom is it the case, that we are called to a patient laboring under "winter fever"---a term very vague and excessively vulgar---until after expectoration is established. Theory, reason and experience, teach us to aid nature in her efforts to relieve the diseased organ. Her remedy, is expectoration; and as Art is only the hand-maid of Nature, we should, in humility, be guided by her, as by a superior. Hence, we should be extremely cautious in the application of any means which could direct her recuperative efforts from their natural channel. We relieve her of an excess of the circulating mass, if such excess should be present; we then unload the first passages of all sources of irritation, by appropriate purges; but when such unloading is effected, the rational practitioner rests satisfied with the administration of the mildest and unirritating laxatives or enemata, for the purpose of conveying off the secretions as they are thrown into the alimentary tube.---The next step seems to be, means to prevent the deposition of inflammatory products into the interstitial or parenchymatous texture of the lung, and into the vesicular tissue.—Two sheet anchors come at once to the aid of the "true helper"---Mercury and Antimony: of the two, the first seems generally the preferable one; more especially where there is the danger of gastro-enteric irritation to be apprehended, which seems usually to be the case in marshy localities. Stokes says, that relapses are more rare after a Mercurial treatment, than after an Antimonial. During the month past it has been my lot to treat a greater number of acute and rapid cases of pneumonia, than for any previous year. It assumed a marked bilious type, all occurring in this town and within a few days from the first appearance. Two cases terminated fatally; one of an enfeebled habit, the other rugged, though ailing for some months previous. Both cases were ushered in with delirium: the first aggravated and persistent. I used the lancet in all cases except in those of two children, which were characterized by a low form of excitement.

In the first few cases, I used Antimony, premising bleeding and judicious purging; but as each case was characterized by bilious vomiting, I failed in every instance, to solicit tolerance. I consequently abandoned it, and continued the Mercury, which had been used in conjunction with the Antimony. To prevent its running off by the bowels, and at the same time to quiet nervous excitement, I combined Pulv. Doveri, S. Morphia, or G. Opii, with the Calomel, changing the proportions as particular indications demanded such. For instances:

℞ Calomel and P. Doveri aa ℈j, in 12 parts; one every two hours, or, ℞ Calomel ℈j, and G. Opii grs. xij in 12 portions. Dose, one every hour until six had been taken; then lengthening the interval to two or three hours; or, ℞ Calomel grs. xij vel grs. xviij, et S. Morphine grs. ½ vel grs. j. Dose, one twelfth part every hour or two.

To insure as speedy a *constitutional* effect of Mercury, after free depletion by the lancet, as possible, I instantly directed Calomel ℈ j. and one or two grs. of Opium, to be taken at once, to be followed by a dose of Castor Oil in six or seven hours, if necessary. By such a combination, I thought I could obviate the nervous depression following free bleeding in enfeebled habits, and facilitate the good effects ultimately manifested by Mercury. The Mercury was persisted in, until the gums indicated its specific effect; which, so soon as established, gave immediate relief to the patient; manifested by a free expectoration, by a gentle and uniform moisture of the surface, freedom from pain, and a speedy recovery. The two fatal cases resisted Mercury. Counter irritants were used, but not until the "blistering period" had set in. S. Quinine, I was led to use in the first fatal case, before the inflammatory excitement showed any disposition to subjection.—The dose did not exceed 2 grs., combined with Calomel and P. Doveri. But I was soon compelled to lay it aside and persist in the use of Mercury; and, for a time, it seemed to exert its beneficial influence, which ceased soon however, notwithstanding the continued use of it. Every case which terminated in recovery, showed no disposition to yield, *until* the gums became sore. Although each one was *Mercurialized*, not a single one was "salivated," in the vulgar acceptation of the term. There is a remarkable feature in all acute inflammations; that Mercury can be borne to an exceeding degree, without exerting the deleterious effects on the constitution, so frequently observed in ordinary fevers, in the treatment of which, it produces such lamentable consequences, by reason of its abuse. Inflammation of the lungs may be so intense in degree, as was preeminently the case in one of my fatal cases, as wholly to resist the specific powers of Mercury. Such cases demand special and energetic treatment, to stay destruction. So soon as arterial excitement had in a great measure subsided, showing the violence of the disease to have been removed; I used *Sulph. Quinine*, with the happiest effects.

I cannot imagine any condition of the lungs, in which inflammation has manifested itself, unless wholly induced by malaria, constituting what with propriety, might be denominated *Phlegmasia Miasmatica*, in which the administration of Quinine could at all be justified, unless thrown in with a view to sedation. For in such quantities alone, can the danger of exciting gastro-enteric irritation be obviated. M. Chomel, informs us, that he had used heroic, or, if you please, *sedative* doses of the remedy, "without having observed either gastric inflammation or any of the unfavorable consequences usually attributed to this medicine."

Even in supposing, or granting such a condition of the lungs as might be called *Phlegmasia Miasmatica*, in which Quinine had been successfully used, could we regard it as any thing more than a mere capillary engorgement? Could it be called else than an obscure *irritation*, coming short of inflammation? To distinguish such a case from pneumonia, which, for practical purposes, were desirable, why not designate it by the name of malarious lung fever? Thereby, not of necessity implying by the term fever, an inflammation, which tends only to confuse.

To administer Quinine successfully, in a given case of "winter fever," the diseased state of the lungs, must be very obscure; but on the contrary, the unmistakable manifestations of malarious agencies, stand out in bold relief, casting the inflammation, if such it be, wholly into the shade.

Cases occur in miasmatic localities, which are well calculated to baffle the most experienced, to detect the real nature of the malady ; and fully susceptible of demonstration only by the most critical diagnostic acumen, which by the by, is possessed only by a minority of the Profession resident in malarious districts.

In ascribing to Cinchona and its preparations such *curative* virtues in pneumonitis, we must never confound pneumatalgia with it. We must not lose sight of the pathognomic features of pneumonia. Never forget, that rigor, followed by violent pain in the chest, and attended by fever, and a *sympathetic* cough, etc., sometimes so perfectly simulates acute inflammation of the lung, as to mislead for the time, the most experienced—that unless the most satisfactory stethescopic signs are elicited, we are wholly in doubt, until the appearance of the characteristic sputa. How guarded, therefore, should we be in receiving into our confidence, a medicine so questionable as is Quinia in pneumonia. And furthermore, we can be none too sceptical, in embracing the conclusions of others concerning so vexed a question, unless given in the most definite and intelligible language.

The opinions of learned authors and "Eastern Physicians" are not to be winked at as mere closet lucubrations, and as such, of no value. Another reason which I have already hinted at, why Quinia should be regarded as a hazardous medicine in pneumonia, is, its tendency to excite the mucous membrane of the first passages, increasing its secretion, and frequently to establish a sub-acute mucous enteritis. Now there is a law well known, that in proportion to such irritation, does the probability of secretion in other tissues—as of the lung—diminish ; thereby endangering the integrity of the lung---if the lung be the organ diseased, as in the present instance we will suppose. For it is highly improper to interrupt a free expectoration. As Quinia is a stimulant to some extent, even in sedative quantities; and since the properties of of malaria seem to coincide exactly with those of that combination of water and carburetted hydrogen which arises from marshes; and since the use of irritants is expresssly forbidden by the highest authorities, in the treatment of internal inflammations, created by carburetted hydrogen, it would seem to be highly improper to administer the medicine in a case of *phlegmasia miasmatica*, unless at certain periods and in certain combinations. For, one can readily comprehend the utility of a judicious combination of Calomel, Opii et Quinine, during certain specified conditions or periods of inflammation; but the obvious and palpable utility of the compound rests none the less on the presence of Calomel, which, by its union with Quinia, has its specific effects accelerated, as is well known.

To conclude, lest I weary and exhaust the patience of the reader: experience has taught us that in the accession of a paroxysmal ague, internal organs are flooded with the circulating mass, giving rise to a more or less persistent capillary erethysm, the continuance of which, depends in a great measure on the force of the recuperative powers of resistance inherent in such organs: That, when there is a disposition to take on a diseased action, the engorgement remains as a cause of increased lesion—a train of inflammatory outburst. Therefore, during the earlier period of such inflammation, it is only to be regarded as a symptom of a malarial fever, and as such, to be treated by local means; whereas the general state of the system demands the employment of such general remedies as are applicable in such cases.

